# Spontaneous Regression of Postoperative Paracentral Posterior Retinal Folds after Vitrectomy for Rhegmatogenous Pseudophakic Retinal Detachment

**DOI:** 10.1055/a-2521-4013

**Published:** 2025-02-27

**Authors:** Amr Saad, Karin Fröse, Gabor Mark Somfai, Matthias Becker

**Affiliations:** 1 Department of Ophthalmology, Stadtspital Zürich Triemli, Zurich, Switzerland; 2 Spross Research Institute, Zurich, Switzerland; 3 Department of Ophthalmology, Semmelweis University, Budapest, Hungary; 4 Department of Ophthalmology, University of Heidelberg, Germany

## Introduction/Background

Posterior retinal folds represent a rare yet potentially severe complication following retinal detachment surgery. Although these have a considerable impact on visual outcomes, they are still underreported in the literature, with an incidence as low as 2.8% or as single case reports 1 . As the practice of primary vitrectomy for retinal detachment repair continues to gain popularity 2 , there is an increasing concern about the incidence of this complication 1 . Retinal folds can result in photoreceptor dysfunction, visual field defects, and metamorphopsia, which have a significant impact on patientsʼ quality of life 3 , 4 , 5 , 6 . This case report presents a unique example of spontaneous regression of a paracentral posterior retinal fold following primary vitrectomy for rhegmatogenous retinal detachment in a pseudophakic eye. This contributes to the limited body of evidence on the natural history and management of this complication.

## History and Signs

**Fig. 1:**
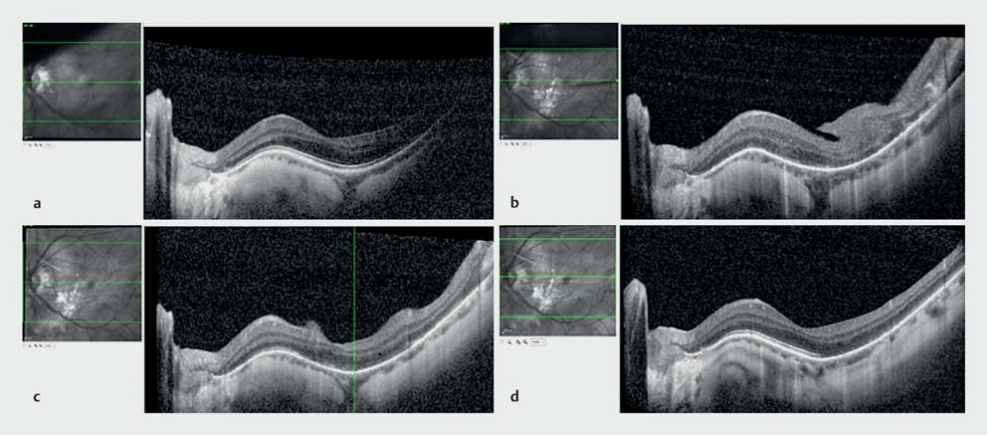
**a** Optical coherence tomography (OCT) of the macula of the left eye, prior to primary vitrectomy, with a superonasal bullous retinal detachment and macula-on. **b** Same OCT section as **a** , 4 days after primary vitrectomy with a paracentral posterior retinal fold involving the temporal macula. **c** The same OCT section as **a** , 39 days after vitrectomy. The fold is already spontaneously regressing, and the macular contour is recognizable, with an intact ellipsoidal zone. **d** Same OCT section as **a** , 88 days after vitrectomy. The macular fold has completely regressed spontaneously.

A 47-year-old male patient with a history of high myopia in the left eye (− 13.75 D sphere with an axial length of 28.4 mm) presented with a superior bullous retinal detachment in his left eye, 13 days after undergoing uneventful phacoemulsification with posterior chamber intraocular lens (IOL) implantation following the diagnosis of advanced cataract. The patient reported a sudden onset of an inferior visual field defect. Fundoscopy revealed a superior bullous macula-on retinal detachment. Optical coherence tomography (OCT) confirmed the macular attachment and the absence of subretinal fluid in the foveal region ( Fig. 1 ).

The patient underwent an uneventful 23-gauge pars plana vitrectomy (PPV) under general anesthesia. The surgical procedure included fluid-air exchange, perfluorocarbon liquid (PFCL) insertion, subretinal fluid drainage, endolaser photocoagulation, and cryotherapy to seal the retinal breaks. The surgery concluded with the implementation of a 20% sulfur hexafluoride (SF6) gas tamponade. The patient was advised to maintain the prone position following the procedure. During the early post-vitrectomy period (4 days postoperatively), as the gas began to absorb, a posterior retinal fold became evident on OCT, extending in the paracentral region ( Fig. 1 ). The patientʼs visual acuity was 20/250.

## Therapy and Outcome

Given the recent reports of spontaneous improvement in some cases of post-vitrectomy retinal folds, a conservative “watch-and-wait” approach was adopted 7 , 8 . The patient was closely monitored with five follow-up visits in the first 3 months, including visual acuity testing, fundus examination, and OCT imaging.

Over the course of approximately 3 months, a gradual and spontaneous regression of the retinal folds was observed. Serial OCT scans demonstrated a progressive flattening of the folds and restoration of the normal retinal contour seen 39 days post-vitrectomy ( Fig. 1 ). Concurrently, the patient reported subjective improvement in visual acuity, with an objective improvement to 20/50, and a concomitant reduction in metamorphopsia.

At 88 days post-vitrectomy, the retinal fold had completely resolved on both clinical examination and OCT imaging ( Fig. 1 ). The patientʼs BCVA had improved to 20/20, with no residual metamorphopsia.

## Discussion

This case demonstrates the potential for spontaneous resolution of posterior retinal folds following vitrectomy for retinal detachment, even in the presence of multiple risk factors for fold formation. The formation of posterior retinal fold risk factors has been linked with multiple risk factors in the literature, including the use of intraocular gas tamponade, recent onset of detachment, and a superior bullous configuration of the detachment. Other contributing factors include large circumferential buckles, external drainage of subretinal fluid, incomplete internal drainage during vitrectomy, and detachment involving the fovea, which creates conditions that facilitate the formation of folds through mechanical and fluid dynamics 1 , 8 , 9 , 10 , 11 .

In our case, the posterior paracentral retinal folds most likely resulted from a combination of factors, such as the gas tamponade, the presence of residual subretinal fluid, and possible retinal slippage during fluid-air exchange 1 , 9 , 11 . The gradual resolution of the fold over a 3-month period indicates that the elasticity of the retina and the potential intrinsic reparative processes of the eye can, in certain cases, overcome the structural alterations induced by fold formation 1 , 12 . It is important to note that the type of retinal fold can influence management decisions 13 . While partial-thickness or outer retinal folds can often be managed conservatively, as seen in our case, full-thickness retinal folds more frequently require surgical intervention, depending on the clinical course and progression 14 .

The current literature lacks standardized treatment recommendations for post-vitrectomy posterior retinal folds. While some case reports recommend prompt surgical intervention, particularly in cases of substantial retinal slippage, others advocate a more conservative approach 5 , 12 . Our case study lends support to the latter strategy, demonstrating that a “wait-and-see” approach can lead to excellent visual outcomes without the need for additional surgical intervention.

In conclusion, this case report underscores the importance of considering conservative management in cases of post-vitrectomy posterior retinal folds due to their potentially benign prognosis, even when multiple risk factors are present. Further research is needed to better understand the natural history of this complication and to develop evidence-based guidelines for its management. Careful patient selection, regular monitoring, and shared decision-making remain crucial in determining the optimal approach for each individual case.
